# The Outcome of Renal Ischemia-Reperfusion Injury Is Unchanged in AMPK-β1 Deficient Mice

**DOI:** 10.1371/journal.pone.0029887

**Published:** 2012-01-09

**Authors:** Peter F. Mount, Kurt Gleich, Shanna Tam, Scott A. Fraser, Suet-Wan Choy, Karen M. Dwyer, Bo Lu, Bryce Van Denderen, Günter Fingerle-Rowson, Richard Bucala, Bruce E. Kemp, David A. Power

**Affiliations:** 1 Department of Nephrology, Austin Health, Melbourne, Victoria, Australia; 2 Kidney Laboratory, Institute for Breathing and Sleep, Melbourne, Victoria, Australia; 3 Protein Chemistry and Metabolism Unit, St. Vincent's Institute, Melbourne, Victoria, Australia; 4 Department of Medicine, University of Melbourne, Melbourne, Victoria, Australia; 5 Immunology Research Centre, St Vincent's Hospital, Melbourne, Victoria, Australia; 6 Department of Internal Medicine, University Hospital Cologne, Cologne, Germany; 7 Department of Medicine, Pathology, and Epidemiology and Public Health, Yale University School of Medicine, New Haven, Connecticut, United States of America; Universidade de Sao Paulo, Brazil

## Abstract

**Aim:**

Activation of the master energy-regulator AMP-activated protein kinase (AMPK) in the heart reduces the severity of ischemia-reperfusion injury (IRI) but the role of AMPK in renal IRI is not known. The aim of this study was to determine whether activation of AMPK by acute renal ischemia influences the severity of renal IRI.

**Methods:**

AMPK expression and activation and the severity of renal IRI was studied in mice lacking the AMPK β1 subunit and compared to wild type (WT) mice.

**Results:**

Basal expression of activated AMPK, phosphorylayed at αThr^172^, was markedly reduced by 96% in AMPK-β1^−/−^ mice. Acute renal ischaemia caused a 3.2-fold increase in α1-AMPK activity and a 2.5-fold increase in α2-AMPK activity (P<0.001) that was associated with an increase in AMPK phosphorylation of the AMPK-α subunit at Thr^172^ and Ser^485^, and increased inhibitory phosphorylation of the AMPK substrate acetyl-CoA carboxylase. After acute renal ischemia AMPK activity was reduced by 66% in AMPK-β1^−/−^ mice compared with WT. There was no difference, however, in the severity of renal IRI at 24-hours between AMPK-β1^−/−^ and WT mice, as measured by serum urea and creatinine and histological injury score. In the heart, macrophage migration inhibitory factor (MIF) released during IRI contributes to AMPK activation and protects from injury. In the kidney, however, no difference in AMPK activation by acute ischemia was observed between MIF^−/−^ and WT mice. Compared with the heart, expression of the MIF receptor CD74 was found to be reduced in the kidney.

**Conclusion:**

The failure of AMPK activation to influence the outcome of IRI in the kidney contrasts with what is reported in the heart. This difference might be due to a lack of effect of MIF on AMPK activation and lower CD74 expression in the kidney.

## Introduction

AMPK is a ubiquitously expressed, energy-sensing kinase that is activated during energy stress by an increase in cellular [AMP] [1]. When activated, AMPK acts to restore energy homeostasis by phosphorylating multiple substrates to both activate pathways of energy production, such as fatty acid oxidation, and to inhibit energy consuming pathways such as protein synthesis and ion transport [2]. AMPK exists as a heterotrimer with a catalytic α subunit and regulatory β and γ subunits [1]. Each of the subunits has multiple isoforms (α1, α2, β1, β2, γ1, γ2, γ3) leading to multiple heterotrimer combinations [2]. In the ischemic heart the effect of AMPK activation is reported to be beneficial by preventing post-ischemic cardiac dysfunction, apoptosis, and injury [3], [4], [5], [6]. These studies, however, have been contradicted by others, which showed that activation of AMPK in the ischemic heart has either no effect [7] or increases apoptosis [8]. Contrasting with the heart, activation of AMPK in the ischemic brain appears to worsen injury [9], [10], [11]. In the kidney, AMPK is reported to be involved in a variety of physiological and pathological processes including ion transport [12], podocyte function [13] and diabetic renal hypertrophy [14]. AMPK is rapidly activated by acute renal ischemia [15] but whether this has an effect on the outcome the outcome of renal IRI is not known.

Stimulation of AMPK in the ischemic heart by macrophage migration inhibitory factor (MIF) is reported to protect against myocardial ischemia-reperfusion injury (IRI) [6], [16]. In contrast, it is unknown whether MIF, which is widely expressed in the normal kidney [17], contributes to AMPK activation by acute renal ischemia.

The present study aims to determine the functional significance of AMPK activation in acute renal ischemia by determining the outcome of IRI in mice deficient for the AMPK β1 subunit (AMPK-β1^−/−^ mice). It also seeks to determine whether MIF contributes to AMPK activation in acute renal ischemia as it does in the heart.

## Materials and Methods

### Materials and reagents

Rabbit polyclonal antibodies against α1-AMPK, α2-AMPK, β1-AMPK, β2-AMPK, γ1-AMPK, γ2-AMPK, pThr^172^α-AMPK and p-ACC-Ser^79^ were produced as previously described [15], [18]. A monoclonal Ab against MIF (ab 7207) was purchased from Abcam (Cambridge, UK). A rabbit monoclonal antibody against ACC1 was from Cell Signaling (MA, USA). A goat polyclonal against CD74 (sc-5438) was purchased from Santa Cruz (CA, USA). Secondary antibodies (swine-anti-rabbit-HRP, rabbit-anti-mouse-HRP) were purchased from Dako (Carpinteria CA, USA). Protein A-HRP was purchased from Amersham Pharmacia (Uppsala, Sweden).

### Animals

AMPK-β1^−/−^ mice were generated on a C57Bl/6 background as recently described [19]. AMPK-α1^−/−^ mice were kindly provided by Professor Benoit Viollet (IC, Institut Cochin Université Paris Descartes). Experiments using the AMPK knockout strains were performed using littermate WT controls from heterozygous mating. The MIF^−/−^ mice used for study are on a C57Bl/6 background, as previously described [20]. Wild type C57Bl/6 mice were from the Walter and Eliza Hall Institute (Parkville, Vic).

### Ethics statement

All animal procedures were performed in the animal facilities of the Austin Hospital or St Vincent's Hospital and were approved by the animal ethics committees of these hospitals. All animal work was conducted according to national and international ethical guidelines.

### Induction of acute renal ischemia

Mice were anaesthetized by intraperitoneal injection of ketamine (85 mg/kg) and xylazine (15 mg/kg). The left kidney was accessed by a midline laparotomy and acute renal ischemia was induced by applying a clamp across the renal pedicle for 10 minutes. The kidneys were rapidly removed and snap frozen in liquid nitrogen for subsequent biochemical analysis. Control kidneys (no ischemia) were removed rapidly without being subject to ischemia.

### Induction of renal ischemia-reperfusion injury

After being anesthetized (ketamine 85 mg/kg, xylazine 15 mg/kg) mice were placed on a heating pad to maintain their core body temperature at 37°C during surgery. A midline abdominal incision was made and the renal pedicles were bluntly dissected. After right nephrectomy, a microvascular clamp (Roboz, Rockville, MD) was placed on the left renal pedicle for either 18 or 20 min while the animal was kept at 37°C in an incubator and well hydrated. The clamp was then removed and the kidney observed to confirm complete reperfusion. The surgical wounds were then sutured in two layers with 5–0 silk. Warm normal saline (100 mL/kg) was instilled into the peritoneal cavity during the procedure. Mice were allowed to recover for 2 hr under a heating lamp and then kept on a heating pad. After 24 hr of reperfusion, the mice were euthanized, blood was taken and kidney samples were obtained for histological analysis.

### Assessment of renal function

Whole blood was collected from an inferior vena cava puncture and sent for biochemical analysis. Serum creatinine level (modified Jaffe rate reaction) and urea level were measured by the Department of Pathology at the St. Vincent's Hospital Melbourne (Olympus AU 2700, Integrated Science, Chatswood, NSW, Australia).

### Preparation of kidney lysates

Lysates of mouse kidney were prepared by homogenization into 2 ml of homogenization buffer (20 mM Tris·HCl, pH 8.0, 2 mM MgCl_2_, 200 mM sucrose, 1% Triton X-100, 50 mM NaF, and protease inhibitors 2 mM PMSF, 1 µM leupeptin, 0.2 µM aprotonin, 1 mM benzamidine, and 1 mM AEBSF) and centrifugation (15,000 *g*, 5 min). The resultant supernatant was further clarified by high-speed centrifugation (75,000 *g*, 30 min).

### Immunoprecipitation of AMPK

AMPK was immunoprecipitated by mixing 1 µl α1-AMPK antibody (1 mg/ml) with 2 mg of kidney lysate at 4°C for 1 hour. Immunocomplexes were precipitated by mixing the sample with Protein A beads (20 µl) (Amersham Pharmacia, Uppsala, Sweden) at 4°C for 30 minutes. After immunoprecipitation the beads were collected by centrifugation and washed 3 times in ice cold wash buffer (1% TritonX-100 in PBS) and once in cold PBS. Reducing Laemmli sample buffer (20 µl) was added to the beads, which were then heated to 95°C for 4 minutes prior to analysis by SDS-PAGE and Western blot.

### Western Blotting

Samples were separated by SDS-PAGE and electrically transferred to a PVDF membrane. The membrane was blocked in 5% casein in Tris-buffered saline (TBS) for 1 hour and then incubated in primary antibody overnight. After washing in TBS 0.05% Tween20 (TBS-T) the membrane was then incubated for 30 minutes in secondary antibody at 1/2500 dilution. After further washing, immunoreactive proteins were detected by enhanced chemiluminescence with the SuperSignal Chemiluminescent System (Perkin Elmer). Western blots were quantified by densitometry (Scion Image for Windows, Scion Corporation, Frederick, Maryland).

### AMPK Activity Assay

AMPK activity was measured as previously described [15]. Briefly, AMPK was immunoprecipitated from kidney lysates and then a phosphorylation reaction was performed in kinase assay buffer [50 mMHepes, pH 7.5, 10 mM MgCl_2_, 5% glycerol, 1 mM DTT, 0.05% Triton X-100, 250 µM (γ-^32^P)-ATP (500 cpm/pmole), 100 µM ADR1 peptide substrate] [21] to measure AMPK activity (pmol/mg/min).

### Renal Histology and Scoring

Ten percent formalin-fixed and paraffin-embedded kidney tissue sections (4 µm) were stained with hematoxylin-eosin. Three high-power fields in each of the cortex, corticomedullary junction and medulla were assessed in a blinded fashion (DP). Scores from the 3 high power fields were then averaged. The degree of tubular necrosis was graded and a modified scoring system was used as previously described [22]: 0, normal kidney; 1, minimal necrosis (<10% involvement); 2, mild necrosis (10%–35% involvement); 3, moderate necrosis (36%–75% involvement); and 4, severe necrosis (>75% involvement).

### Immunohistochemistry

Tissue was immersion-fixed in 4% paraformaldehyde (BDH, Poole, UK), processed, and embedded in paraffin. 4 µm thick paraffin sections were incubated overnight with the anti-MIF antibody (1 µg/ml) at 4°C. Mouse IgG was detected using the mouse peroxidase-antiperoxidase (PAP) technique, comprising a goat anti-mouse Ig (Dako, Carpinteria, CA) diluted at 1/100, followed by mouse PAP (Dako) diluted 1/100. Peroxidase labelling was revealed using the liquid DAB substrate-chromagen system (Dako). Sections were counterstained with hematoxylin.

### Statistics

Statistics were performed using GraphPad Prism Version 4.03 (GraphPad Software, Inc; San Diego, CA). Data are presented as mean ± SD. Unless otherwise stated, data were analyzed by ANOVA; if significant, Bonferroni's test for multiple comparisons was used. P values less than 0.05 were deemed significant.

## Results

### AMPK activation by acute renal ischemia in wild type, AMPK-α1^−/−^ and AMPK-β1^−/−^ mice

**Figure 1:**
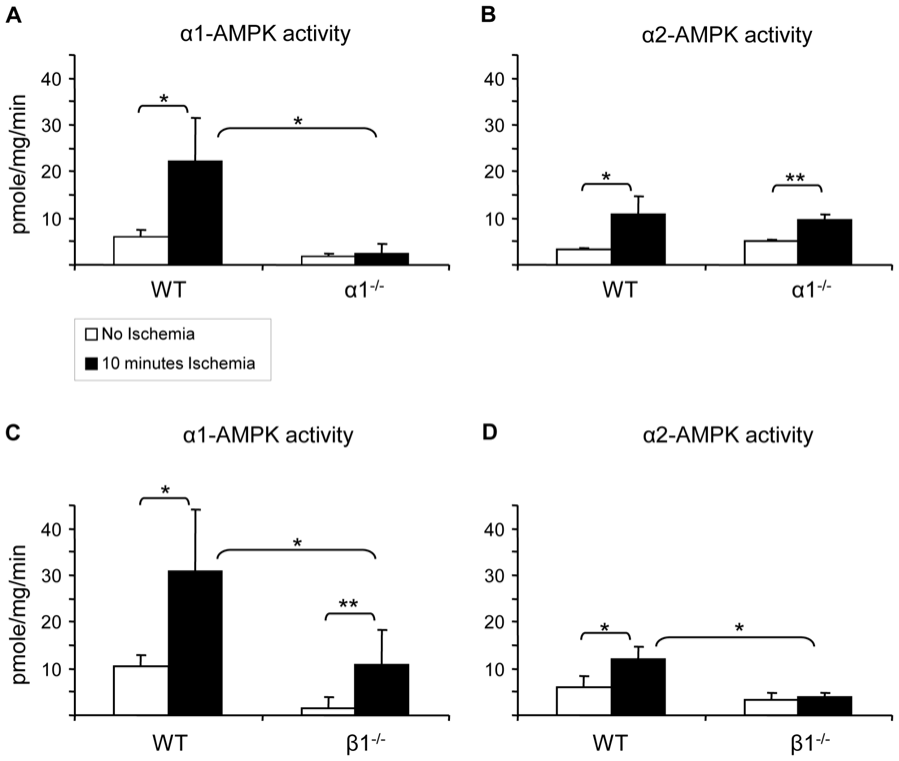
AMPK activation by acute renal ischemia in wild type, AMPK-α1^−/−^ and AMPK-β1^−/−^ mice. Lysates (1 mg protein) from control (□ no ischemia) and ischemic (▪ 10 min ischemia) kidneys of WT (C57Bl/6), AMPK-α1^−/−^ and AMPK-β1^−/−^ mice were immunoprecipitated with antibodies specific for the α1 and α2 AMPK catalytic subunits (n = 6–8 per group). Results are shown as mean ± SD. In WT mice AMPK activity was increased by acute renal ischaemia (P<0.001). In AMPK-β1^−/−^ mice, AMPK activity after acute renal ischaemia was reduced compared to WT for both AMPK-α1 (P<0.001) and AMPK-α2 (P<0.001) isoforms. In AMPK-α1^−/−^ mice there was no activation of AMPK-α1 by acute renal ischemia, whereas AMPK-α2 was activated by acute renal ischemia (P<0.01) by an amount not different to WT. * P<0.001, ** P<0.01.

To determine the potential usefulness of AMPK knockout mice for studying the role of AMPK activation by acute renal ischemia, AMPK activity after renal ischemia was studied in mice deficient for either the α1 or β1 subunit. The phenotypes of the AMPK-α1^−/−^ and AMPK-β1^−/−^ mice have been described previously [19], [23]. Acute renal ischemia was induced by occluding the renal pedicle for 10 minutes and AMPK activity was measured after immunoprecipitations for the α1 and α2 subunits. Acute renal ischemia activated both α1-AMPK (3.2-fold, P<0.001) and α2-AMPK activity (2.5-fold, P<0.001) in WT mice (n = 12–16) (Fig. 1). In kidneys from AMPK-α1^−/−^ mice, basal α1-AMPK activity was reduced by 70% and no increase in α1-AMPK activity was seen with ischemia (Fig. 1A). The observation of residual α1-AMPK activity remaining detectable in AMPK-α1^−/−^ mice indicates some background activity measured by the ADR-1 activity assay that is not accounted for by AMPK, as the AMPK-α1^−/−^ mice are totally deficient for the α1 chain. In kidneys of AMPK-α1^−/−^ mice, α2-AMPK activity and activation by acute ischemia was preserved and not different from WT (Fig. 1B).

In AMPK-β1^−/−^ mice non-ischemic (basal) total (α1 plus α2) AMPK activity compared to WT was reduced from 16.1±4.8 pmol/mg/min to 4.9±3.5 pmol/mg/min (70% reduction, P = 0.001) (Fig. 1C&D). In these mice the reduction in basal α1-AMPK activity was 86% (P<0.001); compared with a 43% reduction for α2-AMPK activity (P = 0.057). α1-AMPK activity after acute renal ischemia was reduced in AMPK-β1^−/−^ mice compared with WT (10.8±7.5 vs 31.0±13.1 pmol/mg/min, 65% reduction, P = 0.002) (Fig. 1C). In addition, α2-AMPK activity was also reduced in AMPK-β1^−/−^ mice after acute renal ischemia compared with WT (3.8±1.0 vs 12.0±2.6 pmol/mg/min, 68% reduction, P<0.001) (Fig. 1D). Whilst AMPK activity was significantly reduced in AMPK-β1^−/−^ mice, in these mice there was still a 7.0-fold relative increase in α1-AMPK activity after acute renal ischemia (P = 0.013) (Fig. 1C). In contrast, for the AMPK-β1^−/−^ mice no increase in AMPK-α2 activity was seen after acute renal ischemia (Fig. 1D).

### Renal expression of activated AMPK in wild type and AMPK-β1^−/−^ mice

**Figure 2:**
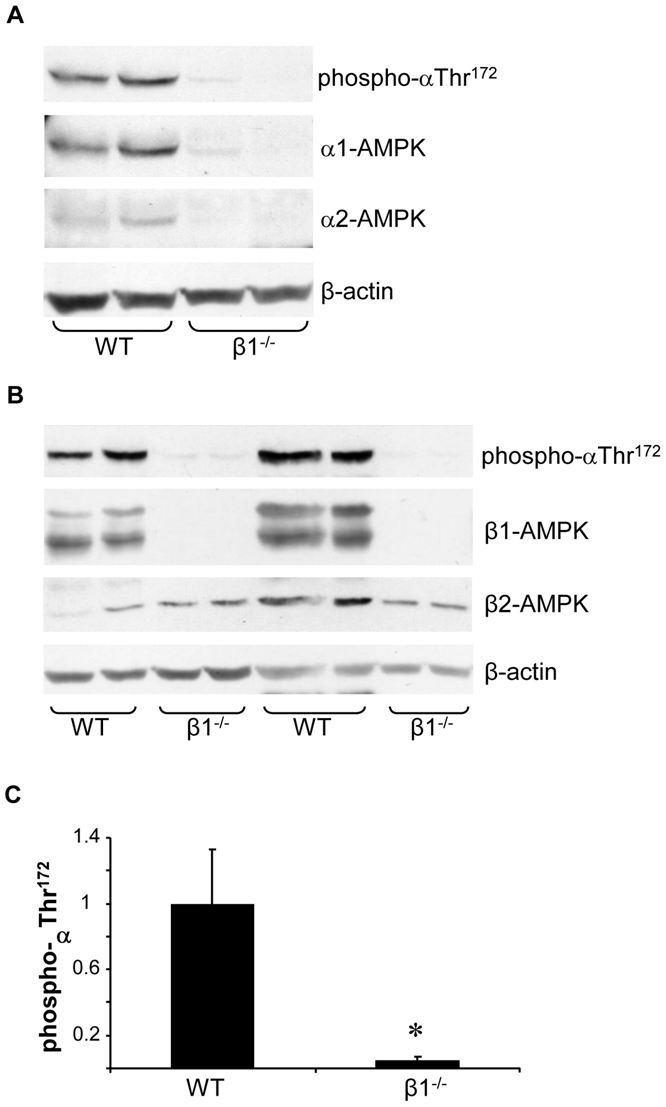
Renal expression of activated AMPK is markedly reduced in AMPK-β1^−/−^ mice. A and B. Lysate from WT and AMPK-β1^−/−^ kidneys were analyzed by Western blot for the activated form of AMPK, phosphorylated at Thr^172^ of the catalytic (α) subunit. The membrane was stripped and reblotted to determine expression of the catalytic (α1 and α2) (A) and scaffolding (β1 and β2) (B) subunits of AMPK. Expression of β-actin was determined to confirm even sample loading. C. Expression of activated (phospho-αThr^172^) in kidneys from WT and AMPK-β1^−/−^ mice was quantified by densitometry using arbitrary units (n = 6 per group). Densitometry for phospho-αThr^172^ was corrected for β-actin. * P<0.001 by unpaired t test.

To further quantify the reduction of renal AMPK activity in AMPK-β1^−/−^ mice, kidney lysates from WT and β1^−/−^ were analyzed by Western blot for expression of the activated form of AMPK, which is phosphorylated at αThr^172^ in the activation loop of the catalytic subunit. In kidneys from AMPK-β1^−/−^ mice, phospho-αThr^172^ expression was profoundly reduced, although not absent (Fig. 2 A & B). By densitometry analysis expression of activated AMPK was reduced by 96% (P<0.001) (Fig. 2C). After probing for phospho-αThr^172^ the membrane was stripped and re-blotted for the α1 and α2 catalytic subunits, which were also both markedly reduced in AMPK-β1^−/−^ mice (Fig. 2A). Western blot analysis for the scaffolding β-subunits confirmed the absence of β1 expression in the AMPK-β1^−/−^ kidney (Fig. 2B). Of note, the β2 subunit isoform was also detectable, and was not different between WT and AMPK-β1^−/−^ (Fig. 2B).

### Severity of renal IRI in wild type and AMPK-β1^−/−^ mice

**Figure 3:**
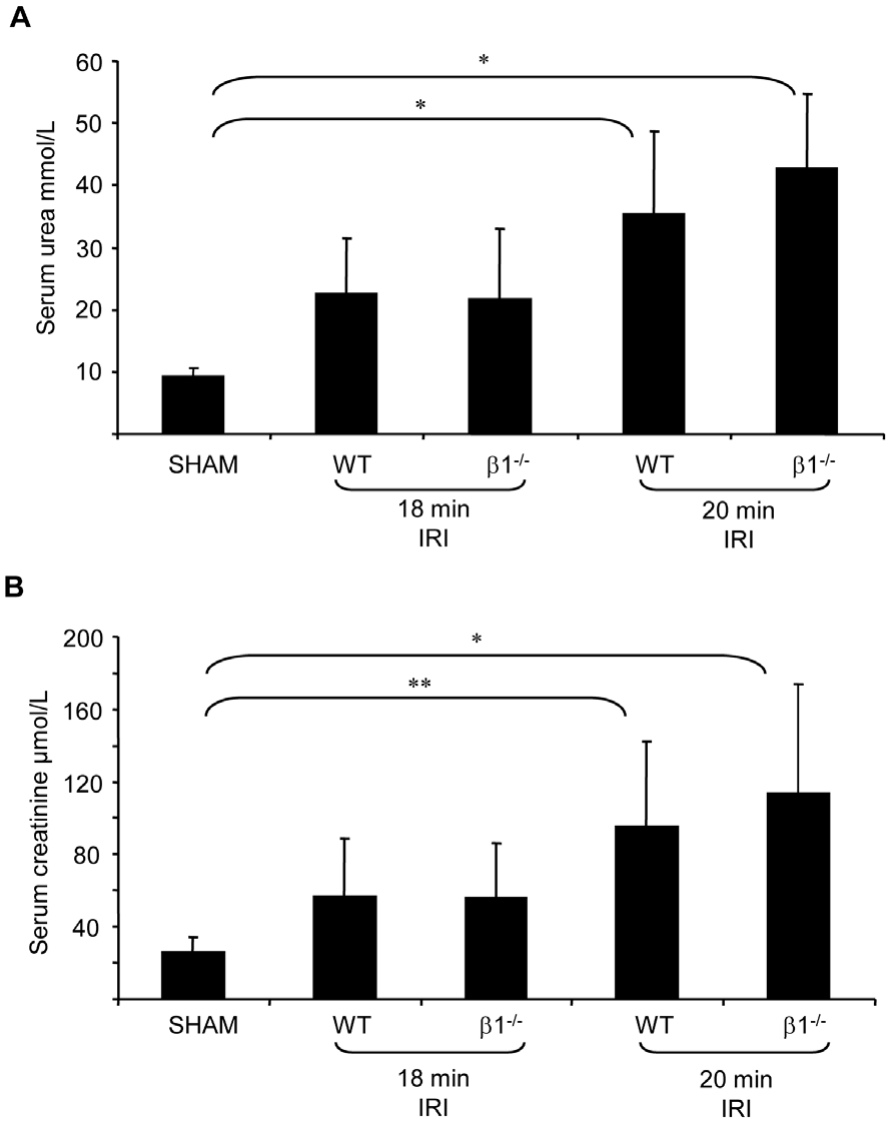
Severity of renal ischemia-reperfusion injury in wild type and AMPK-β1^−/−^ mice assessed by serum urea and creatinine at 24 hours post IR. Serum urea (A) and creatinine (B) were measured 24 hours post renal IR for 18 or 20 minutes in WT and AMPK-β1^−/−^ mice (n = 6–13 per group). Results are shown as mean ± SD. * P<0.001, ** P<0.01. No significant differences between WT and AMPK-β1^−/−^ mice were observed.

Given the significant reductions in α1 and α2 AMPK expression, AMPK activity and activation in the AMPK-β1^−/−^ mice this strain was selected for further study. Unilateral renal IRI was induced in both WT and AMPK-β1^−/−^ mice for 18 or 20 minutes. Control animals underwent a sham procedure. There was no difference in procedure related mortality between the WT and AMPK-β1^−/−^ mice. The severity of renal IRI was determined by measurement of serum urea and creatinine at 24-hours (Fig. 3). After 20 minutes of renal ischemia-reperfusion serum urea and creatinine were increased at 24 hours in both WT and AMPK-β1^−/−^ mice (P<0.001). Importantly, however, no differences in serum urea and creatinine level were observed between WT and AMPK-β1^−/−^ mice.

**Figure 4:**
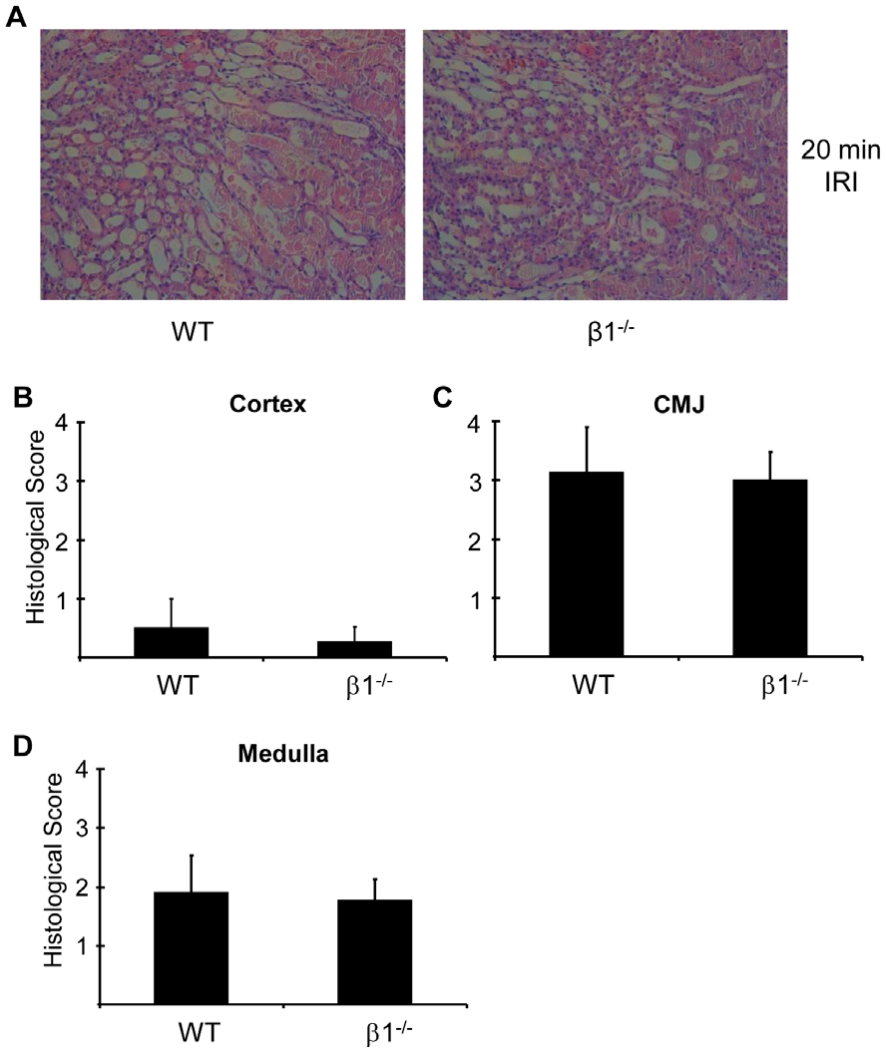
Histological injury 24 hours after renal ischemia-reperfusion injury in wild type and AMPK-β1^−/−^ mice. IRI was performed for 20 minutes and the severity of injury was assessed by histology. A. Histological appearance at 24 hours following 20 mins IRI in WT and β1^−/−^ mice. Images shown are from the region of the corticomedullary junction (CMJ). 200× magnification. The severity of histological injury at 24-hours was quantified in cortex (B), CMJ (C) and medulla (D) as described in methods. No differences were seen between WT and β1^−/−^ mice. n = 7 for WT and 6 for β1^−/−^.

The severity of renal IRI was also assessed by measurement of the histological injury score 24-hours after reperfusion in cortex, corticomedullary junction (CMJ) and medulla (Fig. 4). The more severe injury occurred at the CMJ (Fig. 4C). No differences in the severity of injury were seen between AMPK-β1^−/−^ mice and WT mice.

### AMPK activation and phosphorylation after acute renal ischemia in MIF^−/−^ and wild type mice

**Figure 5:**
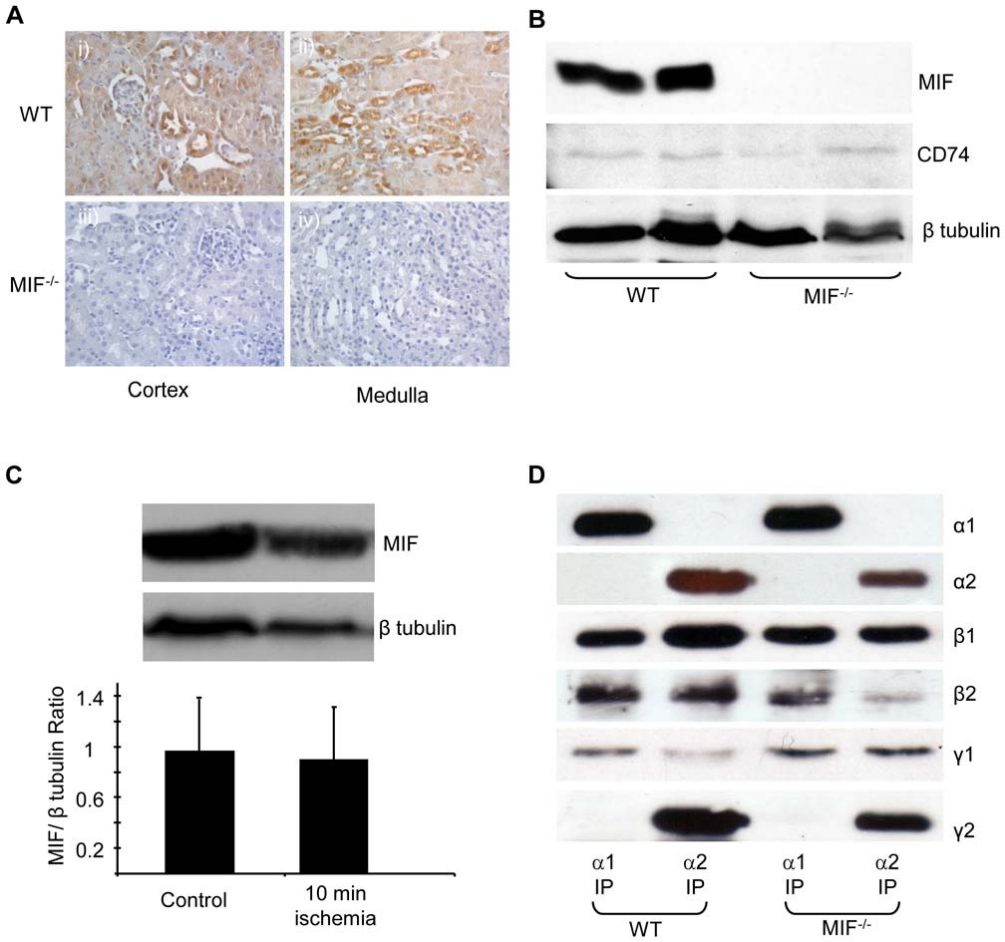
MIF and AMPK expression in MIF and WT mice. A. Renal expression of MIF was determined by immunohistochemical staining using a monoclonal Ab against MIF (ab 7207) in sections from cortex and medulla. To ensure the specificity of staining, immunohistochemistry was performed on sections from WT (C57Bl/6) and MIF^−/−^ kidneys. B. Kidney lysates from MIF^−/−^ and WT mice were analyzed by Western blot with a mouse monoclonal against MIF. MIF was detected as a single band at 14 kDa. There was no difference in CD74 expression between WT and MIF^−/−^ mice. Western blotting for β-tubulin was performed to confirm equivalent protein loading. C. MIF expression was analyzed by Western blot from lysates of control and ischemic (10 mins) kidneys of WT mice. MIF expression was quantified by densitometry of western blot blots control and ischemic kidneys. n = 7 for both groups. MIF expression was normalized to β-tubulin expression to ensure even loading. Results shown as mean ± SD. D. Immunoprecipitations were performed from kidney lysates (4 mg protein) of MIF^−/−^ and WT C57Bl/6 mice with rabbit polyclonal antibodies against the α1 and α2 catalytic subunits of AMPK. The immunoprecipitated complexes were separated by SDS-PAGE and western blotted with antibodies against α1, α2, β1, β2, γ1 and γ2 subunits of AMPK. Blots shown are representative of 3 individually performed experiments.

To determine whether MIF contributes to AMPK activation by acute renal ischemia studies were performed in MIF^−/−^ mice. Immunohistochemical staining confirmed widespread constitutive renal MIF expression, with the predominant site of expression being tubular epithelium (Fig. 5A). The specificity of MIF staining was confirmed by the absence of staining in the MIF^−/−^ mice. Western blot analysis confirmed that MIF was expressed in WT but absent in the kidneys of MIF^−/−^ mice (Fig. 5B). There was no difference in expression of the MIF receptor CD74 between WT and MIF^−/−^ mice (Fig. 5B). Renal MIF expression was unchanged after 10 minutes of acute renal ischaemia (Fig. 5C). There was no difference in AMPK expression or subunit pattern between MIF^−/−^ and WT mice, with the α1, α2, β1, β2, γ1 and γ2 all being expressed (Fig. 5D). The β1 subunit was much more easily detectable than the β2 subunit in the mouse kidney by Western blot, consistent with the earlier finding that β1 is the predominant AMPK scaffolding subunit in the mouse kidney.

**Figure 6:**
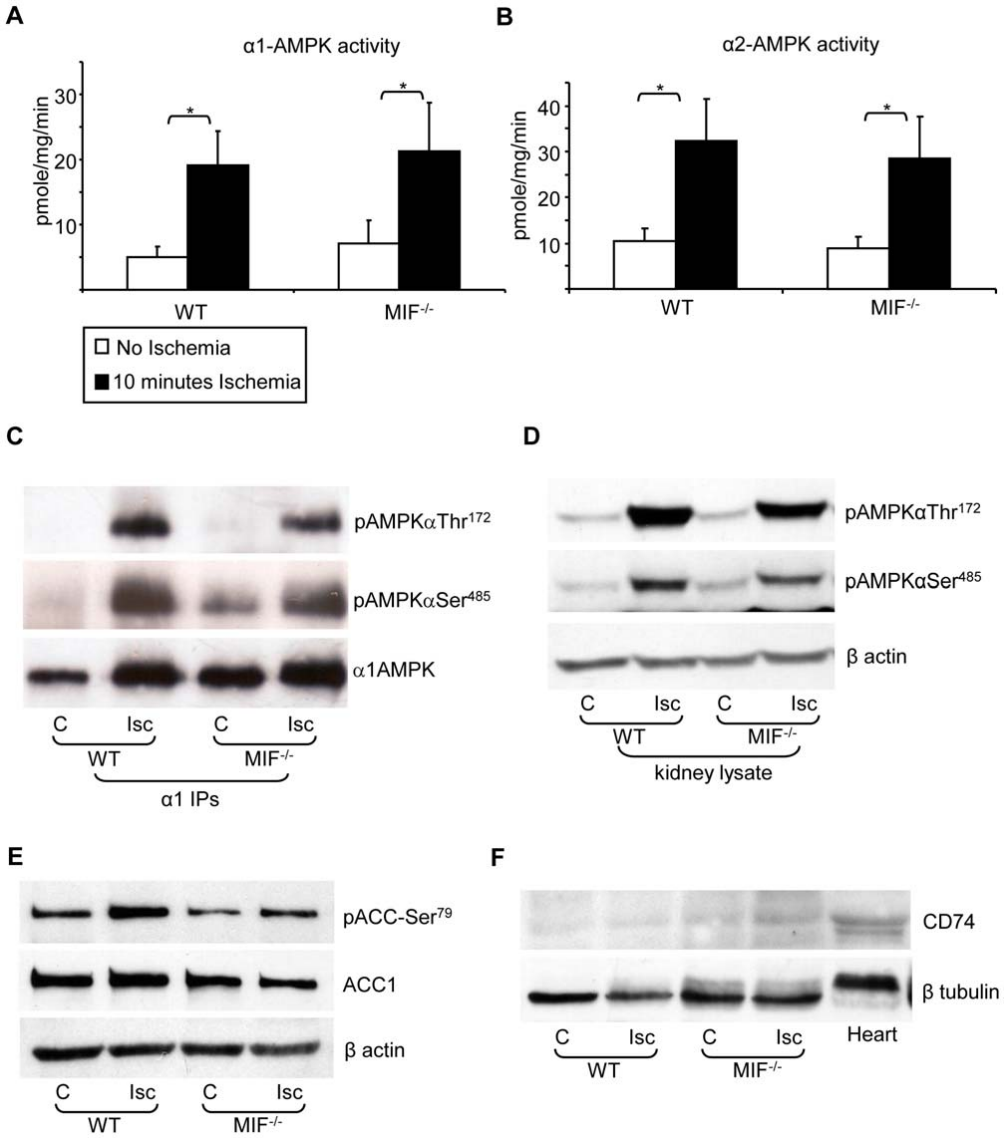
AMPK activation and phosphorylation after acute renal ischemia in MIF^−/−^ mice. Lysates (1 mg protein) from control (□ no ischemia) and ischemic (▪ 10 min ischemia) kidneys of WT (C57Bl/6) and MIF^−/−^ mice were immunoprecipitated with antibodies specific for the α1 (A) and α2 (B) AMPK catalytic subunits (n = 10–12 per group for α1 and n = 6 for α2). AMPK activity was measured by ADR-1 peptide activity assay. Results are shown as mean ± SD. α1-AMPK and α2-AMPK activity were increased several fold after 10 minutes ischemia in WT and MIF^−/−^ mice (* P<0.001). There were no differences in α1-AMPK or α2-AMPK activity between WT and MIF^−/−^ under either control or ischemic conditions. C. Lysates (1 mg protein) from control (C) and ischemic (Isc) kidneys of WT and MIF^−/−^ mice were immunoprecipitated with antibodies specific for the α1 AMPK catalytic subunit and analyzed by Western blotting using antibodies against pAMPKαThr^172^, pAMPKαSer^485^ and α1AMPK (blot shown representative of 4 experiments). D. In addition, whole kidney lysates (50 µg) from the same experiment were separated by SDS-PAGE and analyzed by Western blot for AMPK phosphorylation at αThr^172^ and αSer^485^, with blotting for β-actin as a loading control (blot shown representative of 4 experiments). E. Kidney lysates from this experiment were blotted for inhibitory phosphorylation of ACC at Ser^79^ and expression of total ACC1 (blot shown representative of 4 experiments). F. Heart and kidney lysate was blotted for expression of the MIF receptor CD74.

After 10 minutes of acute ischemia, renal α1-AMPK activity was increased 3.85-fold in WT mice (P<0.001) and 2.79-fold in MIF^−/−^ mice (P<0.001) (Fig. 6A). Similarly, renal α2-AMPK activity was increased by ischemia 3.11-fold in WT mice (P<0.001) and 3.16-fold in MIF^−/−^ mice (P<0.001) (Fig. 6B). There were no differences in renal α1-AMPK or α2-AMPK activity between WT and MIF^−/−^ mice at baseline or after renal ischemia. Activation of AMPK after acute renal ischemia was associated with increased activating phosphorylation of AMPK-αThr^172^ both in WT and MIF^−/−^ with no difference being observed between the two groups (Fig. 6C&D). Interestingly, increased phosphorylation of AMPK-αSer^485^ was also observed in both WT and MIF^−/−^ after acute renal ischemia (Fig. 6C&D), although the role of phosphorylation of AMPK at this site remains unclear [24]. Increased phosphorylation at αThr^172^ and αSer^485^ after acute ischemia was observed both by Western blot of samples immunoprecipitated for α1-AMPK (Fig. 6C) and Western blot of whole kidney lysate (Fig. 6D). Activation of AMPK by acute renal ischaemia in WT and MIF^−/−^ was associated with increased phosphorylation of the AMPK substrate acetyl-CoA carboxylase at Ser^79^ (ACC-Ser^79^) (Fig. 6E), which is known to regulate fatty acid synthesis and oxidation [2]. ACC was observed as a single band with a molecular weight of 250 kDa consistent with the cytoplasmic ACC1 isoform. Since a previous study in the heart has reported MIF to augment ischemic AMPK activation by signaling through CD74 [6], CD74 expression was compared between kidney and heart (Fig. 6F). In the kidney the level of CD74 expression appeared substantially lower than was seen in the heart (Fig. 6F). CD74 expression did not change in the kidney in response to acute ischemia.

## Discussion

The lack of effect of deletion of AMPK-β1 on renal IRI in the present study contrasts with a previous study that found the AMPK activator AICAR, in combination with N-acetylcysteine, attenuated IRI in a canine model of autologous renal transplantation [25]. In that study, however, it is unclear whether the protection against IRI was mediated by AICAR or N-acetylcysteine and also whether any effect of AICAR was mediated by activation of AMPK or other non-specific effects. More recently, Seo-Mayer *et al.* have reported that pre-activation of AMPK before renal IRI by the diabetes drug metformin reduced both histological injury and the level of the urinary biomarker neutrophil gelatinase associated lipocalin (NGAL) [26]. This study also found, consistent with our results, that metformin pre-activation did not affect the increase in serum urea and creatinine observed after renal IRI, suggesting that its effects did not protect against the overall severity of renal injury [26].

Cell culture studies examining the effect of AMPK activation on apoptosis of renal cells in culture have produced variable results. Han *et al.* found that AMPK activation increased hypertonicity-induced apoptosis in medullary interstitial cells [27]. In contrast, Liebarthal *et al* found in cultured proximal tubular cells that activation of AMPK is protective against apoptosis induced by metabolic stress by an Akt dependant mechanism [28]. In fact it is well known that AMPK activation can have either anti-apoptotic or pro-apoptotic effects depending on the cell type and the physiological situation [29]. It is problematic, therefore, to predict from cell culture studies the effect AMPK activation on the severity of kidney injury *in vivo*. The finding of the present study that AMPK activation appeared to have a neutral effect in renal IRI suggest that any possible effect of AMPK on renal cell apoptosis in response to ischaemia appears to be not having an effect on the overall severity of kidney injury and renal dysfunction.

The finding that reduced AMPK activation does not alter the severity of renal IRI contrasts with studies of myocardial ischemia, where activation of AMPK appears protective [3], [6]. Further, activation of AMPK in the ischemic brain appears harmful [10], [11]. Our data suggest a neutral effect of AMPK activation within the kidney in a model of warm IRI. The beneficial effects of AMPK in ischemia have generally been attributed to its effect on energy homeostasis, which act to prevent ATP depletion and subsequent apoptosis [3]. The mechanism by which AMPK activation by ischemia can exacerbate injury, as seen in stroke, is less clear but has been proposed to involve lactic acidosis, glucose transporter upregulation, and activation of autophagy [30]. It has also been proposed that in some circumstances activation of AMPK by myocardial ischaemia can be harmful, possibly by worsening intracellular acidosis [31]. Activation of AMPK in response to ischemia is likely to regulate multiple substrates and pathways, some of which may be protective and others of which may be harmful.

The principle finding of this study is that the severity of IRI was unaltered in AMPK-β1^−/−^ despite the fact that basal AMPK expression, activity and activation were significantly reduced. This suggests that AMPK is unlikely to have a vital protective role in renal IRI. A limitation of the present study, however, is that, while significantly and substantially reduced, a low level of AMPK activity and activation was still detectable in kidneys from AMPK-β1^−/−^ mice. The AMPK activity data estimates a reduction of renal AMPK activity in the AMPK-β1^−/−^ mice of approximately 70%, whereas the Western blot data suggests a more profound 96% reduction in the expression of activated AMPK. This discrepancy might be explained, at least in part, by the observation that the AMPK activity assay does detect a low but measurable level of non-specific background activity that is not altered by the presence or absence of AMPK expression. Interestingly, while AMPK activity was reduced in AMPK-β1^−/−^ mice, the relative level of AMPK activation with ischemia was similar in WT and AMPK-β1^−/−^ mice. The residual AMPK activity and activation in the AMPK-β1^−/−^ can be explained by expression of the AMPK-β2 subunit that was detectable, at a proportionally low level, in the mouse kidney. Interestingly, in the rat kidney β2 has been observed as the predominant AMPK scaffolding subunit [32], [33], contrasting with the findings seen here in the mouse kidney where β1 is predominant. Whilst the results of the present study should be interpreted in the context of the limitation of the model used at present a total AMPK null kidney is not available. This is because total AMPK knockout has been found to be embryonically lethal at day 10.5 post-conception [34]. An additional consideration is the possibility that there are differences in the functions of the various AMPK catalytic or regulatory subunits in the kidney that are presently not known. Further studies in mice with other disruptions to the AMPK pathway might provide further insight into the role of AMPK in renal IRI.

In the present study we observed that AMPK activation by acute renal ischaemia occurred simultaneously with increased phosphorylation of both AMPK-αThr^172^ and AMPK-αSer^485^. Whilst the role of AMPK-αThr^172^ phosphorylation in the activation of AMPK is well-characterized [35], the functional significance of phosphorylation at the AMPK-αSer^485^ site, which exists in the βγ binding domain of the α subunit [36], is not well understood. Whilst two studies have reported that phosphorylation of AMPK-αSer^485^ might in fact be inhibitory [37], [38], this is contradicted by the observation that the activating mutation of this site to aspartate is still robustly activated by liver purified AMPK-kinase [24]. The kinase(s) responsible for AMPK-αSer^485^ have not been extensively studied, although both insulin and hepatitis C have been reported to inhibit AMPK activation by mediating phosphorylation of this site by Akt/PKB [37], [38]. In addition however, AMPK-αSer^485^ is reported to be phosphorylated by liver purified AMPK-kinase, but whether this was mediated by LKB1, CaMKKβ or autophosphorylation was not defined [24]. These apparent inconsistencies might be explained by differences in the sequences of phosphorylation events. For example, in the study by Horman *et al.* where AMPK-αSer^485^ appears inhibitory, phosphorylation of AMPK-αSer^485^ preceded phosphorylation of AMPK-αThr^172^. In contrast, in the present study in which AMPK-αSer^485^ phosphorylation appears to occur either after or simultaneous to AMPK-αThr^172^ phosphorylation, a marked increase in AMPK activity was seen despite the fact that phosphorylation at AMPK-αSer^485^ was increased.

The finding in the present study that the presence of MIF did not contribute to activation of AMPK in the ischemic kidney contrasts with what has been reported in the ischemic heart and cultured cardiomyocytes [6], [16]. We also did not observe any effect of recombinant MIF on AMPK activity in a kidney cell line (data not shown). The receptor for MIF has been identified as CD74, which is the MHC class II invariate chain [39]. Furthermore, signaling through CD74 appears to mediate MIF induced activation of AMPK in the heart [6]. Sanchez-Nino *et al.* have reported constitutive CD74 expression in the rat kidney, which was localized to glomeruli and tubules and increased with diabetes [40]. Furthermore, CD74 was found to be expressed by cultured podocytes and proximal tubular cells [40]. In the present study we found to that CD74 was detectable by Western blot of kidney lysates, but the level of expression was markedly lower than in the heart. This observation raises the possibility that the lower CD74 expression seen in the kidney compared to the heart might help explain the different effects of MIF on AMPK activity between heart and kidney. At present, however, the explanation for the difference between the regulation of AMPK by MIF between the heart and the kidney is not clear. Nonetheless, it is known that activation of AMPK in response to specific regulators can vary between different tissues. For example AMPK activity is increased by leptin in skeletal muscle [41] and reduced by leptin in the hypothalamus [42]. The two major pathways of AMPK activation are known to be mediated by an increase in cellular [AMP], acting via the upstream kinase LKB1 [43], or an increase in cellular [Ca ^2+^], acting via the upstream kinase CaMKKβ [44]. Whilst acute renal ischemia is known to acutely increase both cellular [AMP] and [Ca^2+^] [45], it is not known whether activation of AMPK by acute renal ischemia occurs predominantly downstream of LKB1 or CaMKKβ. The present study shows, however, that the pathways of AMPK activation by acute renal ischemia involve phosphorylation of AMPK-αThr^172^ by upstream kinase(s) and this is not influenced by the presence or absence of MIF.

In summary, this study found that AMPK is activated in response to acute renal ischemia and that activation is associated with phosphorylation of AMPK at αThr^172^ and αSer^485^ and inhibitory phosphorylation of ACC-Ser^79^. Significant constitutive expression of MIF in tubular epithelium was found, but there was no difference in AMPK activation by acute renal ischemia between WT and MIF^−/−^ mice. Expression of AMPK and activation of AMPK by acute renal ischemia was markedly attenuated in AMPK-β1^−/−^ mice. Despite this there was no difference in the severity of renal IRI, as measured biochemically or histologically, between AMPK-β1^−/−^ and WT mice. The lack of effect of reduced AMPK activation on the outcome of renal IRI suggests that renal protection in situations of severe energy stress is unlikely to be a critical function of AMPK in the kidney. This supports a more general view that the primary role of AMPK in the kidney is likely to be to detect and respond to more minor perturbations of cellular energy status that occur within the context of normal renal physiology.
